# Effectiveness of Ultrasound-guided versus Landmark-based Glucocorticoid Injection in the Treatment of First Carpometacarpal Joint Osteoarthritis 

**DOI:** 10.24908/pocus.v8i2.16594

**Published:** 2023-11-27

**Authors:** Shamma Ahmad Al-Nokhatha, Sinead Maguire, Luke Corcoran, Neil Mac Eoin, Richard Conway, Ciaran Johnson

**Affiliations:** 1 Department of Rheumatology, St. James's Hospital Dublin Ireland; 2 Department of Rheumatology, Tawam Hospital Al Ain United Arab Emirates; 3 College of Medicine and Health Sciences, UAE University Al Ain United Arab Emirates; 4 Department of Radiology, St. James's Hospital Dublin Ireland

**Keywords:** ultrasonography, hand, osteoarthritis, corticosteroid

## Abstract

**Background: **Osteoarthritis is a debilitating degenerative disease more pronounced in elderly affecting many joints. The first carpometacarpal joint (CMC1) is commonly affected. Pain is the major complaint, which can impact patient’s daily activities. Intra-articular glucocorticoid injection can be considered if conservative measures fail and ultrasound guided injection might be superior to the traditional anatomic landmark-guided technique. **Objective: **The aim of this study is to evaluate the effectiveness of ultrasound-guided versus landmark-based approach to intra-articular CMC1 injection using the Australian Canadian osteoarthritis hand index (AUSCAN). **Methods: **Adult patients diagnosed with symptomatic CMC1 osteoarthritis who failed conservative measures were enrolled. In this prospective observational cohort study, utilizing a convenience sample, intra-articular corticosteroid injection was administered either by ultrasound-guided technique or landmark-based approach. Pain, stiffness and function in 10-points scale at baseline, 6 and 12 weeks were collected and analyzed using descriptive analysis. **Results: **There were 33 patients enrolled. Mean age was 63 years, with females making up the majority of participants (n = 28, 84.8%). Mean duration of CMC1 pain was 10 months (SD=2.5) up to the point of receiving the injection. Ultrasound guided injection was performed in 60.6% (n=20), while 39.4% (n=13) had the landmark approach. Both groups achieved a statistically and clinically significant level of change in AUSCAN score at week 6 (P≤ 0.05) but with a recurrence of symptoms at week 12 (P ≤ 0.05). At both intervals the AUSCAN scores were better than baseline (P ≤ 0.05). There was no difference between the two groups regarding baseline pain VAS score (mean ultrasound group= 6.6 vs landmark group= 7.5; P = 0.18). No significant differences were identified between two groups in terms of changes from baseline to 6, 12 and between 6 to 12 weeks in pain, stiffness and hand function (P > 0.05). **Conclusion: **No difference was found between the ultrasound-guided and landmark-based approaches for CMC1 injection on pain score, stiffness, or function.

## Background

**Figure 1:**
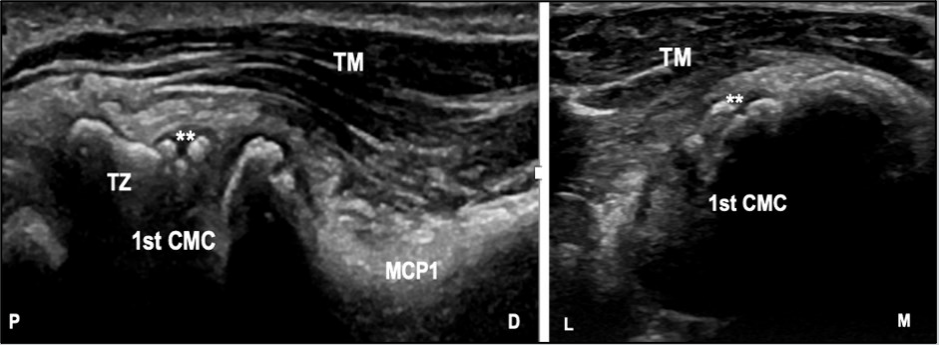
Transverse (right) and longitudinal (left) scans of the left first CMC demonstrating osteophytes **. Thenar muscle (TM), First metacarpal bone (MCP1), Trapezium (TZ), L: lateral, M: medial, P, proximal, D, distal.

Osteoarthritis (OA) is a debilitating degenerative disease that affects many joints. The carpometacarpal is one of the commonly affected sites (Figure 1). Pain in particular is a major component and this can significantly impact patients’ activities of daily living 1, 2. The prevalence of symptomatic thumb OA as recorded by the Framingham study is 2.7% and 5% for males and females, respectively. More recent studies have reported a prevalence of erosive OA of the first CMC (CMC1) of 2.2% 3, 4. According to the recent 2019 American College of Rheumatology and Arthritis Foundation guidelines, in patients who fail non-pharmacological management, medication can be considered, among which intra-articular glucocorticoid injection is noted to be more efficacious in comparison to other compounds. Ultrasound (US) guidance can facilitate the accuracy at the target point, which may improve outcomes 5. The aim of this study is to evaluate the effectiveness of ultrasound-guided compared to landmark-based intra-articular injection of CMC1. 

## Methods

A prospective, observational cohort study was conducted at St James’s hospital in Ireland between January and July 2021. The study employed a convenience sample, comprising patients from the dedicated patient lists of two expert doctors who routinely perform therapeutic injections. Ethical approval was obtained from the St James’ and Tallaght Hospital Joint Ethics Committee. To be included the following inclusion criteria was required of all participants: adults (aged ≥18 years) diagnosed with symptomatic first CMC osteoarthritis with Eaton-Littler stage 2 and greater^6^ who had failed conservative measures such as anti-inflammatory treatment and occupational therapy, with or without splint usage). Those who had received a glucocorticoid injection in the past three months were excluded. All injections consisted of 20mg of depomedrone with a local anesthetic (0.5 ml 1% lidocaine). One of two techniques were performed: the landmark approach, which involved identifying the anatomical landmarks for the carpometacarpal joint, and the ultrasound-guided technique (using GE Logiq P9 machine). Demographic, smoking history and employment data were collected at the time of injection. Participants completed the Visual Analogue Scale (VAS) for pain severity assessment (range: 0-10) and the Australian Canadian Osteoarthritis Hand Index (AUSCAN) questionnaire, which assessed hand pain, stiffness and function on a scale of 0 to 10. This questionnaire was completed by all participants at baseline, 6 weeks and 12 weeks, which aligns with the average duration of injection’s effect 7, 8.

### Statistical analysis

Data were entered into JASP software and then were analyzed. The efficacy of the two methods were compared based on the pain severity, stiffness, and function of the patient using t-tests and ANOVA. The relation between qualitative variables was assessed using chi-squared tests.

## Results

There were 33 patients enrolled in this study. The mean age was 63 years (SD = 9.5). There was a higher proportion of females (84.8%, n = 28) compared to males. At the time of injection majority (81.8%, n = 27) were unemployed, and (72.7%, n = 24) were non-smokers. One patient was left-handed (3%) while the rest were right-handed (n=32, 97%). The mean duration of CMC1 pain was 10 months (SD = 2.5). There was no difference in baseline pain VAS scores between the two groups (mean US group= 6.6 vs landmark group= 7.5; P = 0.18).

Over one third of the participants (36.4%, n = 12) did not use a hand splint and, 45.5% (n= 15) had a previous glucocorticoid injection to the joint of interest. The majority of patients (69.7%, n = 23) had a right CMC1 glucocorticoid injection. Overall, 60.6% (n= 20) had ultrasound-guided injection and 39.4% (n = 13) had a landmark-based injection. 

**Figure 2:**
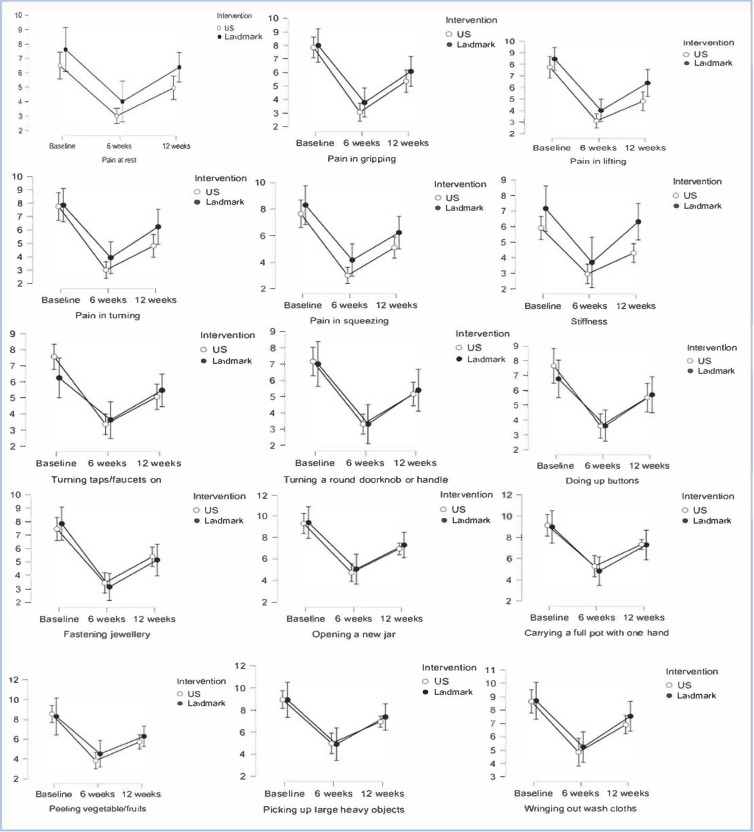
Changes in Australian Canadian osteoarthritis hand index (AUSCAN) scores at week 6 with a recurrence of symptoms at week 12 in the study groups ultrasound (US) vslandmark based injection technique.

Both groups achieved a statistically significant improvement in AUSCAN score at week 6 (P≤0.05) but with a recurrence of symptoms at week 12 (P ≤0.05), at both intervals the AUSCAN scores were better than baseline (P ≤0.05) (Figure 2). No significant differences were identified between two groups in terms of changes from baseline to 6, 12, and between 6 to 12 weeks in pain, stiffness and hand function. The US-guided injection group and the standard approach group had significant better results in pain score at rest, gripping, lifting, turning, squeezing, and stiffness in 6 weeks’ time as highlighted in Table 1. In the US-guided injection group hand function improved significantly at weeks 6 and 12 (P<0.05) in all aspects assessed apart from carrying a full pot with one hand (P= 0.06). While in the landmark-based approach group significant improvements were captured at week 6 (P <0.05) (Table 1). No injection side reactions or complications were reported during the duration of this study.

**Table 1:** Comparison changes between two groups (ultrasound-guided vs landmark-based approach) in CMC1 pain, stiffness and function.

-	**Group**	**Mean difference**	**Std. deviation**	**P value**	**P value (US vs landmark)**
Pain at rest (Vas score)	US	3.5	0.599	**<0.001**	0.094
Landmark	2.5	0.879	**0.047**	-
**Change after 12 weeks**	US	1.55	0.599	0.084	-
-	Landmark	0.115	0.879	0.896	-
**Change between 6-12 weeks**	US	-1.95	0.599	**0.02**	-
-	Landmark	-2.385	0.743	**0.021**	-
-					
Pain in gripping	US	4.8	0.547	**<0.001**	0.373
Landmark	4.23	0.678	**<0.001**	-
**Change after 12 weeks**	US	2.5	0.547	**<0.001**	-
-	Landmark	1.92	0.678	**0.037**	-
**Change between 6-12 weeks**	US	-2.3	0.547	**<0.001**	-
-	Landmark	-2.3	0.678	**0.008**	-
-					
Pain in lifting	US	4.65	0.542	**<0.001**	0.109
Landmark	4.462	0.673	**<0.001**	-
**Change after 12 weeks**	US	2.95	0.542	**<0.001**	-
-	Landmark	2.077	0.673	**0.018**	-
**Change between 6-12 weeks**	US	-1.7	0.542	**0.018**	-
-	Landmark	-2.385	0.673	**0.006**	-
-					
Pain in turning	US	4.75	0.607	**<0.001**	0.197
Landmark	3.923	0.753	**<0.001**	-
**Change after 12 weeks**	US	2.95	0.607	**<0.001**	-
-	Landmark	1.615	0.753	0.214	-
**Change between 6-12 weeks**	US	-1.8	0.607	**0.03**	-
-	Landmark	-2.308	0.753	**0.026**	-
-					
Pain in squeezing	US	4.65	0.615	**<0.001**	0.153
Landmark	4.154	0.762	**<0.001**	-
**Change after 12 weeks**	US	2.55	0.615	**0.001**	-
-	Landmark	2.077	0.762	0.059	-
**Change between 6-12 weeks**	US	-2.1	0.615	**0.009**	-
-	Landmark	-2.077	0.762	0.059	-
-					
Stiffness	US	2.95	0.581	**<0.001**	0.124
Landmark	3.462	0.721	**<0.001**	-
**Change after 12 weeks**	US	1.6	0.581	0.07	-
-	Landmark	0.846	0.721	1	-
**Change between 6-12 weeks**	US	-1.35	0.581	0.188	-
-	Landmark	-2.615	0.721	**0.007**	-
-					
Turning taps/faucets on	US	4.2	0.538	**<0.001**	0.751
Landmark	2.615	0.667	**0.003**	-
**Change after 12 weeks**	US	2.5	4.651	**<0.001**	-
-	Landmark	0.769	0.667	0.759	-
**Change between 6-12 weeks**	US	-1.7	0.538	**0.024**	-
-	Landmark	-1.846	0.667	0.067	-
-					
Turning around doorknob or handle	US	3.85	0.576	**<0.001**	0.964
**Change after 6 weeks**	Landmark	3.692	0.715	**<0.001**	-
**Change after 12 weeks**	US	2	0.576	**0.01**	-
-	Landmark	1.615	0.715	0.192	-
**Change between 6-12 weeks**	US	-1.85	0.576	**0.021**	-
-	Landmark	-2.077	0.715	**0.046**	-
-					
Doing up buttons	US	4.05	0.651	**<0.001**	0.742
**Change after 6 weeks**	Landmark	3.154	0.808	**0.003**	-
**Change after 12 weeks**	US	2.15	0.651	**0.018**	-
-	Landmark	1.077	0.808	0.822	-
**Change between 6-12 weeks**	US	-1.9	0.651	**0.049**	-
-	Landmark	-2.077	0.808	0.113	-
-					
Fastening jewellery	US	4	0.557	**<0.001**	0.947
**Change after 6 weeks**	Landmark	4.602	0.691	**<0.001**	-
**Change after 12 weeks**	US	2.05	0.557	**0.005**	-
-	Landmark	2.296	0.891	0.074	-
**Change between 6-12 weeks**	US	-1.95	0.557	**0.008**	-
-	Landmark	-2	0.691	**0.042**	-
-					
Opening a new jar	US	4.55	0.603	**<0.001**	0.691
**Change after 6 weeks**	Landmark	4.308	0.748	**<0.001**	-
**Change after 12 weeks**	US	2.35	0.603	**0.003**	-
-	Landmark	2.077	0.748	**0.044**	-
**Change between 6-12 weeks**	US	-2.2	0.603	**0.005**	-
-	Landmark	-2.231	0.748	**0.033**	-
-					
Carrying a full pot with one hand	US	3.85	0.651	**<0.001**	-
**Change after 6 weeks**	Landmark	4.154	0.808	**<0.001**	-
**Change after 12 weeks**	US	1.8	0.651	0.06	-
-	Landmark	1.692	0.808	0.241	-
**Change between 6-12 weeks**	US	-2.05	0.651	**0.028**	-
-	Landmark	-2.462	0.808	**0.034**	-
-					
Peeling vegetables/fruits	US	4.7	0.633	**<0.001**	0.607
**Change after 6 weeks**	Landmark	3.769	0.785	**<0.001**	-
**Change after 12 weeks**	US	2.8	0.633	**<0.001**	-
-	Landmark	2	0.785	0.082	-
**Change between 6-12 weeks**	US	-1.9	0.633	**0.039**	-
-	Landmark	-1.769	0.785	0.139	-
-					
Picking up large heavy objects	US	3.95	0.615	**<0.001**	0.879
**Change after 6 weeks**	Landmark	4	0.762	**<0.001**	-
**Change after 12 weeks**	US	2	0.615	**0.02**	-
-	Landmark	1.538	0.762	0.239	-
**Change between 6-12 weeks**	US	-1.95	0.615	**0.021**	-
-	Landmark	-2.462	0.762	**0.02**	-
-					
Wringing out wash cloths	US	3.8	0.611	**<0.001**	0.645
**Change after 6 weeks**	Landmark	3.462	0.757	**<0.001**	-
**Change after 12 weeks**	US	1.75	0.611	**0.051**	-
-	Landmark	1.154	0.757	0.664	-
**Change between 6-12 weeks**	US	-2.05	0.611	**0.015**	-
-	Landmark	-2.308	0.757	**0.034**	-

## Discussion

Osteoarthritis involving the first CMC is a degenerative joint condition for which patients seek intervention to alleviate pain and improve hand function. In this study, 33 participants with symptomatic CMC1 arthritis were prospectively evaluated for response to either ultrasound-guided or landmark-based joint injection. Both cohorts were predominantly female, with a median age of 65 years consistent with baseline demographics of previous studies 5, 7, 9. Previous research has shown that intra-articular corticosteroid injections can improve hand pain and function regardless of osteoarthritis stages. However, they have also been shown to be more efficacious for longer durations (beyond 3 months) for early (Eaton 1-2) than late (Eaton 3-4) osteoarthritis stages 9, 2, similar to the outcome of using hand splint 10. 

In addition, a case series of 43 patients with grade 2 or higher Eaton classification were assessed following ultrasound-guided injection to the CMC1 and a strong correlation was found between patients who had persistent pain following glucocorticoid and local anesthesia injection at one week and the progression to surgery, (odd ratio 3.1) 11. Factors influencing the likelihood of a repeat injection or surgical intervention are beyond the scope of this study.

Several studies have been done comparing use of ultrasound-guided versus landmark-based approach in small joint or structures 12, 13, 14. To et al. performed a similar study on cadavers, they found that the success rate by injection site was higher for ultrasound-guided participants than for non-ultrasound participants for thumb CMC arthrosis (72% vs. 38%), which was confirmed by fluoroscopy and later by dissection and localizing the blue dye mix in the cadavers 12. On the other hand, Derian et al. found no statistically significant difference in accuracy between the two methods (ultrasound vs. landmark approach) in cadaver investigations 15, although no clinical conclusion could be drawn from these studies.

In this study, we found that the effect of intra-articular corticosteroid injection is satisfactory but transient for both ultrasound-guided and landmark based CMC1 injection

## Limitations

Our study had limitations, including a small sample size, convenience sample of doctor’s own patients, lack of blinding, and two experts performing the injection in both arms.

## Conclusion

In a small study of ultrasound versus landmark based CMC1 injection for OA, both techniques provided similar transient pain relief.

## Ethics approval and consent to participate

 Ethical approval was obtained from the St James’ and Tallaght Hospital Joint Ethics Committee, Written informed consent was obtained from all individual patients included in the study.

## Availability of data and material

The data supporting the study's findings are available from the corresponding author upon a reasonable request.

## Competing interests

The authors declare that they have no competing interests. 

## Funding

No author received funding for this study. 

## Authors’ contributions

SAA: data collection, data analysis, manuscript writing. SM: data collection and manuscript writing. LC and NME: data collection. RC and CJ: manuscript writing/editing. All authors read and approved the final manuscript.
